# Prostate cancer cells modulate osteoblast mineralisation and osteoclast differentiation through Id-1

**DOI:** 10.1038/sj.bjc.6605480

**Published:** 2009-12-15

**Authors:** H-F Yuen, Y-T Chiu, K-K Chan, Y-P Chan, C-W Chua, C M McCrudden, K-H Tang, M El-Tanani, Y-C Wong, X Wang, K-W Chan

**Affiliations:** 1 Department of Pathology, The University of Hong Kong, Pokfulam, Hong Kong, China; 2 Department of Anatomy, The University of Hong Kong, Pokfulam, Hong Kong, China; 3 Center for Cancer Research and Cell Biology, Queen's University of Belfast, Belfast, UK

**Keywords:** Id-1, bone metastasis, prostate cancer, osteoblast, osteoclast

## Abstract

**Background:**

Id-1 is overexpressed in and correlated with metastatic potential of prostate cancer. The role of Id-1 in this metastatic process was further analysed.

**Methods:**

Conditioned media from prostate cancer cells, expressing various levels of Id-1, were used to stimulate pre-osteoclast differentiation and osteoblast mineralisation. Downstream effectors of Id-1 were identified. Expressions of Id-1 and its downstream effectors in prostate cancers were studied using immunohistochemistry in a prostate cancer patient cohort (*N*=110).

**Results:**

We found that conditioned media from LNCaP prostate cancer cells overexpressing Id-1 had a higher ability to drive osteoclast differentiation and a lower ability to stimulate osteoblast mineralisation than control, whereas conditioned media from PC3 prostate cancer cells with Id-1 knockdown were less able to stimulate osteoclast differentiation. Id-1 was found to negatively regulate TNF-*β* and this correlation was confirmed in human prostate cancer specimens (*P*=0.03). Furthermore, addition of recombinant TNF-*β* to LNCaP Id-1 cell-derived media blocked the effect of Id-1 overexpression on osteoblast mineralisation.

**Conclusion:**

In prostate cancer cells, the ability of Id-1 to modulate bone cell differentiation favouring metastatic bone disease is partially mediated by TNF-*β*, and Id-1 could be a potential therapeutic target for prostate cancer to bone metastasis.

Prostate cancer is the second leading cause of cancer-related death in men in the United States (Jemal *et al*, 2008). Patients with localised prostate cancer have a high 5-year survival rate (Klotz, 2005; Walz *et al*, 2007). However, once metastatic disease is detected, the survival rate is greatly reduced (Sabbatini *et al*, 1999; Loberg *et al*, 2005). Bone is the predominant site of distant metastasis from prostate cancer (Coleman, 2006; Guise *et al*, 2006; Vessella and Corey, 2006). Recent studies on inhibition of osteolytic prostate cancer to bone metastasis have given promising results on suppression of the ability and extent of metastases establishment (Saad *et al*, 2002, 2004).

The importance of Id-1 in the metastatic progression of various types of cancer has been shown (Sikder *et al*, 2003; Wong *et al*, 2004; Ling *et al*, 2006). In prostate cancer, Id-1 has been shown to promote cancer progression through various mechanisms. Increased Id-1 expression was detected during carcinogenesis of the prostate in the Noble rat (Ouyang *et al*, 2001). In human specimens, Id-1 expression in prostate cancer is increased in comparison to benign prostatic hyperplasia specimens (Ouyang *et al*, 2002a), and increased expression of Id-1 is correlated with Gleason score, reflecting the aggressive nature of Id-1-positive prostate cancers (Ouyang *et al*, 2002a; Coppe *et al*, 2004; Yuen *et al*, 2006). This information suggests that Id-1 is important in both prostate cancer initiation and progression. Delineation of the molecular mechanisms of Id-1 in prostate carcinogenesis and cancer progression has recently begun. Id-1 promotes prostate cancer growth through inactivation of the p16/pRB pathway (Ouyang *et al*, 2002b), and activation of the epidermal growth factor receptor (Ling *et al*, 2004) and the MAPK pathway (Ling *et al*, 2002). Id-1 promotes survival of prostate cancer cells through the NF-*κ*B pathway (Ling *et al*, 2003), and it regulates the apoptotic response of cancer cells towards various chemodrugs (Zhang *et al*, 2007b), such that inactivation of Id-1 results in increased sensitivity of prostate cancer cells to paclitaxel through the JNK pathway (Zhang *et al*, 2006). Most importantly, Id-1 has been shown to promote metastasis of prostate cancer. Id-1 promotes angiogenesis through upregulation of VEGF (Ling *et al*, 2005), while it also binds to caveolin-1 to induce epithelial–mesenchymal transition in prostate cancer cells (Zhang *et al*, 2007a). In addition, increased expression of Id-1 is significantly associated with shorter survival of prostate cancer patients (Forootan *et al*, 2007). Taken together, these results suggest that Id-1 has important roles in prostate cancer carcinogenesis and metastatic progression.

Id-1 also functions to regulate the differentiation of osteoblasts. Id-1 has been shown to regulate the expression of osteocalcin, an osteoblast-differentiation marker (Tamura and Noda, 1994). Overexpression of Id-1 inhibits osteogenic differentiation induced by bone morphogenetic proteins (BMPs) (Peng *et al*, 2004). In an *in vivo* breast cancer to bone metastasis model, the presence of BMP-2 in a scaffold increased the metastatic frequency of the breast cancer cell line SUM1315 (Moreau *et al*, 2007). On the other hand, treatment with BMP-2 in the breast cancer cell line C2C12 resulted in increased expression of Id-1 (Katagiri *et al*, 1994; Nobta *et al*, 2005; Raida *et al*, 2005; Langenfeld *et al*, 2006), while BMP-2 also positively regulated the expression of Id-1 in certain cell contexts (Locklin *et al*, 2001; Takeda *et al*, 2004). Because BMPs have very important roles in the development of bone metastasis in prostate cancer cells (Feeley *et al*, 2005, 2006), it might be possible that BMP-2 in the bone environment promotes metastasis of cancer cells to bone through upregulation of the intrinsic expression of Id-1 in cancer cells. BMP-2 increases the invasiveness of prostate cancer cells (Dai *et al*, 2005). Increased expression of BMP-7 has been detected in prostate cancer to bone metastases, and high-level expression of BMP-7 is related to osteoblastic activity of the metastatic lesion (Masuda *et al*, 2003). BMP-6 is highly expressed in prostate cancer to bone metastases (Autzen *et al*, 1998), and a high level of BMP-6 in prostate cancer cells promotes osteoblastic activity of bone cells (Dai *et al*, 2005). In a recent study, overexpression of BMP-6 was shown to be correlated with increased Id-1 expression, suggesting that Id-1 might work downstream of BMP-6 in promoting prostate cancer progression (Darby *et al*, 2008). Overall, these results imply that Id-1 has a role in prostate cancer to bone metastasis.

Previously, we have shown that Id-1 expression in primary prostate cancer is correlated with Gleason score (Ouyang *et al*, 2002a; Yuen *et al*, 2006), and in a separate cohort we also showed that it is also correlated with mortality (Forootan *et al*, 2007). In this study, we aimed to identify the role of Id-1 in prostate cancer to bone metastasis and its possible downstream targets by studying how bone cells respond to conditioned medium from prostate cancer cells expressing various levels of Id-1.

## Materials and methods

### Cell culture conditions

Human prostate cancer cell lines LNCaP and PC3 (American Type Culture Collection (ATCC)) were maintained in RPMI-1640 (Invitrogen, Carlsbad, CA, USA) supplemented with 5% FBS (Invitrogen). An organ-confined prostate cancer cell line 22RV1 (from Professor Frankie Chan) and a human osteosarcoma cell line SaOS-2 (ATCC) were maintained in RPMI-1640 supplemented with 10% FBS. A murine macrophage cell line RAW264.7 (ATCC) was maintained in DMEM (Invitrogen) supplemented with 10% FBS. LNCaP-pBabe-puro or pBabe-Id-1 stable transfectants were generated previously (Ling *et al*, 2003), and were maintained in RPMI-1640 supplemented with 5% FBS with intermittent selection with puromycin (0.5 *μ*g ml^–1^). A PC3 stable transfectant containing pLentiviral-shId-1 and the control PC3 pLentiviral-siCon were generated as previously described using lentiviral-mediated transduction (Cheung *et al*, 2006).

### Plasmid

Id-1 expression plasmid, pcDNA-Id-1 was a gift from Dr Eiji Hara (Kyoto Prefectural University of Medicine, Kyoto, Japan). pGL3-IL6pro was generated by cloning of the IL-6 promoter region, amplified using genomic DNA from PC3 cells and primers IL-6-ProF1-Mlu1 (5′-TATACGCGTCACTCCACCTGGAGACGCCT-3′) and IL-6-ProR1-Bgl2 (5′-GCCAGATCTGAGTTCATAGCTGGGCTCCT-3′), into pGL3 (Promega, Madison, WI, USA). pGL3-TNF*β*pro was generated by cloning of the TNF-*β* promoter region, amplified using genomic DNA from PC3 cells and primers TNF*β*-ProF1-Mlu1 (5′-TATACGCGTGAAGCCTGTACTCAGCCAAGG-3′) and TNF*β*-ProR1-Bgl2 (5′-GCTAGATCTCGGTAGTCCAAAGCACGAAG-3′) into pGL-3. Amplification of these promoter regions was performed by using the high-fidelity PCR System (Roche Molecular Biochemicals, Indianapolis, IN, USA) and sequencing was performed to confirm correct sequence of the insert. pLentiviral vector containing a short hairpin interfering RNA (shRNA) sequence against Id-1 mRNA (5′-AACTCGGAATCCGAAGTTGGA-3′) (Zheng *et al*, 2004) was generated using the BLOCK-iT lentiviral RNAi Expression System (Invitrogen). pLentiviral vector control containing shRNA sequence (5′-GCGTATTGCCTAGCATTAC-3′), which has no significant homology to any coding sequences in the human genome, was also generated.

### Western blotting

Western blotting was performed as previously described (Kwok *et al*, 2005). Id-1 (C-20) antibody was from Santa Cruz Biotechnology Inc. (Santa Cruz, CA, USA) and was used in a 1 : 1000 dilution. IL-6 antibody from Research & Diagnostics System Inc. (Minneapolis, MN, USA) was used at a concentration of 5 *μ*g ml^–1^. TNF-*β* antibody from Abcam Inc. (Cambridge, MA, USA) was used in a 1 : 1000 dilution. Actin antibody from Sigma-Aldrich (St Louis, MO, USA) was used in a 1 : 3000 dilution.

### Reverse-transcription–PCR

Reverse transcription–PCR was performed as previously described (Ling *et al*, 2005). Primer sequences were as follows; IL-6F1 (5′-CTGGATTCAATGAGGAGACTTGC-3′) and IL-6R1 (5′-GGACAGGTTTCTGACCAGAAG-3′); TNF*β*-F1 (5′-CTCCCCATGACACCACCTGAACGTC-3′) and TNF*β*-R1 (5′-CTACAGAGCGAAGGCTCCAAAGAAG-3′); ActinF1 (5′- GTGGGGCGCCCCAGGCACCA-3′) and Actin R1 (5′-CTCCTTAATGTCACGCACGATTTC-3′).

### Luciferase reporter assay

Luciferase reporter assay was performed as previously described (Ling *et al*, 2005).

### Collection of conditioned medium

6.25 × 10^5^ cells (LNCaP and PC3) were seeded in T-25 flasks. After 24 h attachment and proliferation, the medium was replaced with RPMI-1640 or DMEM containing 0.5% FBS, respectively. The medium was collected after another 48 h, filtered and stored at −80 °C before use. In the rescue experiments, the conditioned medium from LNCaP pBabe Id-1 cells was supplemented with human recombinant TNF-*β* protein (Abcam) to 100 pM, the concentration previously shown to stimulate another human osteosarcoma cell line MG-63 (Thomson *et al*, 1987). In the case of PC3, the conditioned medium from PC-3 shId-1 cells was supplemented with a TNF-*β* antibody (Abcam) to 2 ng ml^–1^, the concentration recommended by the manufacturer for neutralisation.

### *In vitro* osteoclastogenesis assay

The conditioned medium from both cell types was mixed with DMEM-10% FBS in a 1 : 1 ratio. Control was a mixture of 50% RPMI-1640 and 50% DMEM-10% FBS. The conditioned medium was then supplemented with 50 ng ml^–1^ RANKL (R&D Systems). 5 × 10^3^ RAW264.7 cells were seeded into each well of a 96-well plate and were allowed to grow for 24 h. Medium was then replaced with the mixed conditioned medium after every 3 days. After an 8-day treatment with the mixed conditioned medium, the number of differentiated osteoclasts was determined using TRACP staining assay kit according to the manufacturer's instructions (Sigma). A red-stained cell with three or more nuclei was counted as a differentiated osteoclast-like cell. The total number of osteoclast-like cells was counted in the entire well.

### *In vitro* osteoblast mineralisation assay

The conditioned medium from both cell types was mixed with RPMI-1640 10% FBS in a 1 : 1 ratio. RPMI-1640 was used instead of conditioned medium as control. The mixed conditioned medium was then supplemented with 10 mM *β*-glycerophosphate and 50 *μ*g ml^–1^ ascorbic acid (both from Sigma). 5 × 10^3^ SaOS-2 cells were seeded into each well of a 24 well-plate. The culture medium was changed every third day. On day 9, the medium was replaced with the mixed conditioned medium and this medium was changed every third day. On day 21, cells were stained for calcium deposition by alizarin red S staining.

### Alizarin red S staining

Cells were air-dried for 10 min, fixed in 50% ethanol at room temperature for 10 min thrice. The cells were then stained with 10 mg ml^–1^ alizarin red S for 5 min and were then washed with 1 × PBS. The staining extent was recorded by photography and the retained dye was then extracted by extraction solution (20% methanol and 10% acetic acid in water). The absorbance at 450 nm of the extracted solution was then measured.

### Patients and specimens

A total of 110 archival formalin-fixed paraffin-embedded prostate cancer specimens were obtained from the Department of Pathology, The University of Hong Kong. These specimens were incorporated into tissue microarray as previously described (Yuen *et al*, 2006). Needle biopsy specimens used previously were not included in this study because of insufficient tissue for immunohistochemical staining. Specimens were collected consecutively excluding those from patients who had received previous treatment directed against prostate cancer or those with insufficient tissue for tissue microarray incorporation. Details of patient data are listed in Table 1.

### Immunohistochemistry

Immunohistochemical staining was performed as previously described using EnVision+ system-HRP (Dako, Carpinteria, CA, USA) (Yuen *et al*, 2007). A monoclonal antibody of IL-6 (R&D Systems) was used at a concentration of 2.5 *μ*g ml^–1^ whereas polyclonal antibody of TNF-*β* (Abcam) was used in a dilution of 1:500.

### Quantification of immunohistochemical staining results

Evaluation was carried out as previously described (Yuen *et al*, 2007). In brief, the extent and intensity of the staining were graded by an arbitrary scale ranging from 0 to 3 representing negative (0), weak (1), moderate (2) and strong (3) staining. Negative and weak staining was classified as low-level whereas moderate and strong staining was classified as high-level expression. The number of cases varies slightly because of the difference in interpretable cores in tissue microarray.

### Statistical analysis

Statistical analysis was performed using SPSS 15.0 software (SPSS Inc, Chicago, IL, USA). Differences in expression level between groups/samples were analysed using chi-square, Fisher's exact test or Mann–Whitney-*U* tests where applicable. Spearman's rank test was applied to test correlations of the expression levels between different proteins, and correlations of the expressions of different proteins and Gleason grade. The association between the expression level and the risk of developing distant metastasis was estimated using Kaplan–Meier analysis and compared using log-rank test.

## Results

### Overexpression of Id-1 in osteoblastic LNCaP cells suppressed their ability to stimulate osteoblast mineralisation and promoted their ability to stimulate osteoclast differentiation

**Figure 1:**
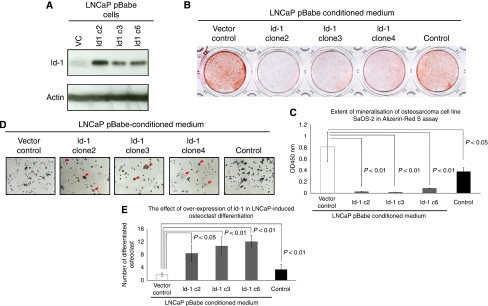
The effects of Id-1 overexpression in LNCaP prostate cancer cells in prostate cancer cell-mediated bone cell activities. Three independent clones of Id-1 overexpressing cells previously generated were used in this study. (**A**) Western blot analysis shows increased Id-1 protein expression compared with vector control. (**B**) Alizarin red S assay; conditioned medium from LNCaP-Id-1 clones inhibited mineralisation of SaOS-2 osteosarcoma cells, whereas conditioned medium from LNCaP-vector control cells stimulated mineralisation of SaOS-2 osteosarcoma cells when compared with control. (**C**) Quantitative analysis of the extent of mineralisation. (**D**) Conditioned medium from LNCaP-Id-1 clones stimulated the differentiation of RAW264.7 cells to osteoclast-like cells, whereas medium from LNCaP-vector control conferred no significant effect when compared with control. Representative area under × 200 magnification. (**E**) Quantitative analysis of the osteoclast differentiation assay. Columns represent the mean values from three independent experiments±s.d.

LNCaP cells (which express low levels of Id-1) were engineered to ectopically express high levels of Id-1 by retroviral transduction. Three clones (namely, clones 2, 3 and 6) with high levels of Id-1 expression were generated previously (Ling *et al*, 2002). These clones also have higher MAPK pathway activity and PSA expression than vector control clones (Ling *et al*, 2002, 2004). Using western blot analysis, these three clones were confirmed as expressing higher levels of Id-1 than vector control in culture medium supplemented with 0.5% serum (Figure 1A). To analyse whether overexpression of Id-1 modulates prostate cancer cell-mediated bone cell differentiation, conditioned medium from LNCaP pBabe vector control cells and Id-1 overexpressing cells were collected and used for treating a human osteosarcoma cell line, SaOS-2, which is capable of undergoing mineralisation, and also a mouse macrophage-like cell line RAW264.7, which is capable of differentiating into osteoclast-like cells upon appropriate stimulation. As shown in Figure 1B and C, SaOS-2 cells underwent mineralisation after treatment with 10 mM *β*-glycerophosphate and 50 *μ*g ml^–1^ ascorbic acid. Conditioned medium from LNCaP pBabe vector control cells increased the extent of mineralisation, as shown by alizarin red S assay. When quantitatively analysed, the degree of mineralisation of SaOS-2 cells treated with conditioned medium from LNCaP pBabe vector control cells (Figure 1C, open column) was significantly higher (*P*<0.05) than the negative control (without prostate cancer conditioned medium, black-filled column). These results suggest that conditioned medium from LNCaP cells could stimulate the mineralisation of human osteosarcoma SaOS-2 cells. However, when SaOS-2 cells were treated with conditioned medium from LNCaP cells expressing high levels of Id-1, mineralisation was severely inhibited (Figure 1B and C). The extent of mineralisation detected by retained alizarin red S dye was significantly lower in SaOS-2 cells treated with conditioned medium from LNCaP cells overexpressing Id-1 (grey columns, *P*<0.05 for each of the three clones, compared with either vector control conditioned medium or control medium). These results suggest that ectopic expression of Id-1 inhibits LNCaP-induced osteoblast mineralisation.

As shown in Figure 1D and E, treatment of RAW264.7 cells with control medium resulted in only 3.3±1.5 differentiated osteoclast-like cells per well (Figure 1E, black-filled column). The number of differentiated osteoclast-like cells was similar (1.7±0.6, *P*>0.05) when RAW264.7 cells were treated with conditioned medium from LNCaP pBabe vector control cells, suggesting that conditioned medium from LNCaP cells is incapable of stimulating osteoclast differentiation. However, when RAW264.7 cells were treated with conditioned medium from LNCaP pBabe Id-1 cells, the number of differentiated osteoclast-like cells was significantly higher (clone 2: 8.3±2.5, clone 3: 10.7±2.9 and clone 6: 12±2, grey columns, *P*<0.05 for each the three clones compared with either vector control conditioned medium or control medium). These results suggest that ectopic expression of Id-1 in LNCaP cells increases their ability to stimulate osteoclast differentiation. Taken together, these results provide evidence that Id-1 expression in LNCaP results in reduced ability to stimulate osteoblast activity and increased ability to stimulate osteoclast activity.

### Downregulation of Id-1 in the osteolytic prostate cancer cell line PC3 reduces its ability to stimulate osteoclast differentiation

**Figure 2:**
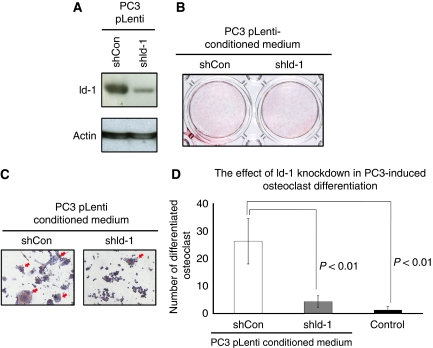
The effects of Id-1 knockdown on PC3-mediated bone cell activities. (**A**) PC3 shId-1 cells were generated by lentiviral transduction and the reduction in Id-1 expression was confirmed using western blot analysis. (**B**) Conditioned media from both PC3 shCon and shId-1 cells conferred inhibitory effects on SaOS-2 mineralisation, indicating that knockdown of Id-1 is not sufficient to modulate the ability of PC3 cells to stimulate osteoblast mineralisation. (**C**) Conditioned medium from PC3 shId-1 cells had a lower ability to stimulate osteoclast differentiation when compared with that from PC3 shCon cells. (**D**) Quantitative analysis of osteoclast differentiation in PC3. Columns represent the mean values from three independent experiments±s.d.

We analysed whether the knockdown of Id-1 in prostate cancer cells would also affect their ability to mediate bone cell activities. Using lentiviral infection with shRNA, we established a stable clone of the osteolytic PC3 prostate cancer cell line (which endogenously expresses high levels of Id-1) that expressed low levels of Id-1. As shown in Figure 2A, the Id-1 protein level was significantly reduced in PC3 pLenti-shId-1 when compared with control PC3 pLenti-shCon cells. SaOS-2 cells mineralised when cultured in osteogenic medium containing both *β*-glycerophosphate and ascorbic acid, as shown using alizarin red S assay (Figure 1B and C). When the conditioned media from these two cell lines were applied to SaOS-2 cells, we found that mineralisation of SaOS-2 cells was inhibited regardless of the expression level of Id-1 (Figure 2B). These results suggest that reduction of Id-1 expression does not affect PC3-inhibited osteoblast mineralisation. On the other hand, reduced Id-1 expression in PC3 cells led to significant reduction of its ability to induce osteoclast differentiation (*P*<0.05). As shown in Figure 2C and D, treatment of RAW264.7 with conditioned medium from PC3 pLenti shCon cells resulted in 26.3±8.3 osteoclast-like cells per well, whereas only 1.3±1.2 osteoclast-like cells were observed when RAW264.7 cells were treated with control medium (open column *vs* black-filled column). In addition, when RAW264.7 cells were treated with conditioned medium from PC3 pLenti shId-1 cells, only 4.3±2.1 osteoclast-like cells were observed per well (grey-filled column). These results suggest that conditioned medium from PC3 cells induces macrophage to osteoclast differentiation, and that silencing Id-1 in osteolytic PC3 prostate cancer cells inhibits the ability of their conditioned medium to induce osteoclast differentiation.

### Id-1 negatively regulates the expression of TNF-*β*

**Figure 3:**
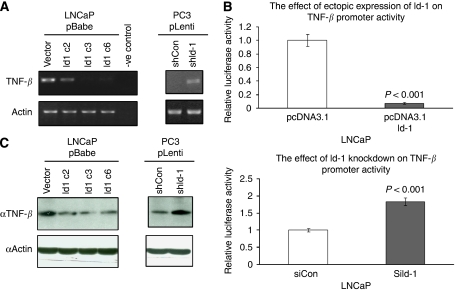
Id-1 negatively regulates TNF-*β* expression. (**A**) Using RT–PCR, we found that the expression of TNF-*β* mRNA was lower in Id-1 overexpressing LNCaP cells and was higher in Id-1 knockdown PC3 cells compared with respective controls. Water was used in place of template in the negative control. (**B**) Id-1 negatively regulated the promoter activity of TNF-*β*, suggesting that the regulation might be at the transcriptional level. (**C**) Western blot showed similar results at the protein level. Columns represent the mean values from three independent experiments±s.d.

To analyse whether downstream factors of Id-1 are responsible for its pro-osteolytic effect in prostate cancer cells, we used RT–PCR to test the expression of several secretory factors in prostate cancer cells expressing various levels of Id-1. We found that TNF-*β* mRNA expression was reduced in LNCaP overexpressing Id-1 compared with LNCaP vector control cells, whereas it was increased in PC3 cells expressing low levels of Id-1 compared with PC3 vector control cells (Figure 3A). We went on to study whether Id-1 could modulate the promoter activity of TNF-*β*. Using PCR, we amplified an ∼500-bp upstream region of TNF-*β* and cloned it into pGL-3 (Promega) for promoter luciferase reporter assay. We found that the expression level of Id-1 was negatively correlated with TNF-*β* promoter activity in LNCaP cells, such that increased Id-1 by overexpression reduced the promoter activity, whereas reduced Id-1 by siRNA knockdown enhanced the promoter activity (Figure 3B). These results suggest that Id-1 negatively regulates TNF-*β* promoter activity. To analyse whether the protein level of TNF-*β* was affected by Id-1, we also performed western blotting for protein extracted from LNCaP and PC3 cells expressing various levels of Id-1. As shown in Figure 3C, overexpression of Id-1 in LNCaP cells resulted in reduced protein expression of TNF-*β* whereas knockdown of Id-1 in PC3 cells resulted in increased TNF-*β* protein expression. Overall, these results suggest that Id-1 might negatively regulate TNF-*β* at the transcriptional level.

### Id-1 was negatively correlated with TNF-*β* in human prostate cancer specimens

**Figure 4:**
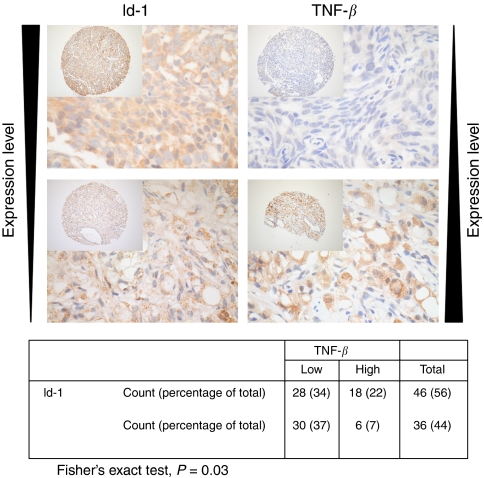
Representative tissue cores of immunohistochemical staining. Id-1 is significantly negatively correlated with TNF-*β* expression in human prostate cancer specimens. A cross-tabulation of Id-1 and TNF-*β* expression in the patient cohort is shown below the immunohistochemistry images.

To test whether the regulatory relationship between Id-1 and TNF-*β* can be observed in clinical materials, we studied whether expression level of Id-1 is correlated with TNF-*β* expression in human prostate cancer specimens using immunohistochemistry in a prostate tissue microarray described previously (Yuen *et al*, 2006). As shown in Figure 4, high-level expression of Id-1 was significantly associated with a low-level expression of TNF-*β* in the prostate cancer specimens (Fisher's exact test, *P*=0.03). However, expression of TNF-*β* did not significantly correlate with Gleason score (chi-square, *P*=0.321) or development of distant metastases (Kaplan–Meier, *P*=0.310). These results suggest that Id-1 might regulate TNF-*β* in primary prostate cancer, although a larger sample size would be needed to determine the relationship between Id-1, TNF-*β* and metastasis. Id-1 has previously been shown to correlate with Gleason score. In this study, we also correlated Id-1 and TNF-*β* with the Gleason score of the tumours and Gleason grade of individual cores. We found that although Id-1 was again significantly correlated with Gleason grade of individual cores in our cohort (Table 2a, *r*=0.356, *P*<0.001), TNF-*β* did not correlated with either Gleason score of the tumours (*r*=−0.028, *P*=0.709) or Gleason grade of the individual cores (Table 2b, *r*=−0.04, *P*=0.532).

### Id-1 modulates prostate cancer-mediated bone cell activity partially through TNF-*β*

**Figure 5:**
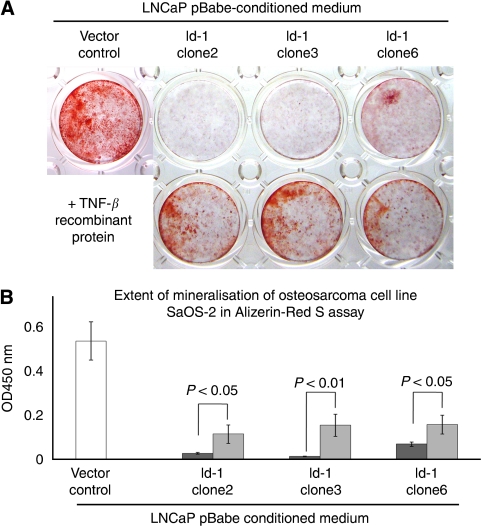
Recombinant TNF-*β* protein rescued the effect of Id-1 overexpression in prostate cancer-mediated osteoblast mineralisation. (**A**) Alizarin red S assay shows that TNF-*β* partially reversed the inhibitory effect of overexpressing Id-1 in LNCaP-mediated osteoblast mineralisation. (**B**) Quantitative analysis of the extent of mineralisation in alizarin red S assay. Light and dark grey columns in bar chart represent results from conditioned medium with and without the addition of TNF-*β* recombinant protein, respectively. Columns represent the mean values from three independent experiments±s.d.

Tumour necrosis factor-*β* has been shown to be expressed in osteoblast-like cells (Bilbe *et al*, 1996). It has also been shown to have a role in stimulating osteoblast proliferation (Frost *et al*, 1997) and osteoblast-mediated osteoclast differentiation (Thomson *et al*, 1987). In this study, we found that prostate cancer cells expressing different levels of Id-1 have both modulated levels of TNF-*β* and an ability to stimulate osteoblast mineralisation and osteoclast differentiation. Therefore, we hypothesised that Id-1 regulates the expression of TNF-*β* that leads to differential ability to stimulate bone cell activities. To test this hypothesis, recombinant TNF-*β* protein was added to LNCaP-Id-1-derived conditioned medium whereas TNF-*β* antibodies were added to PC3-shId-1-derived conditioned medium. As shown in Figure 5, addition of TNF-*β* recombinant protein to LNCaP-Id-1-derived conditioned medium resulted in an increased ability of the medium to stimulate osteoblast mineralisation. This result suggests that suppression of TNF-*β* in LNCaP-Id-1 cells might be responsible for the reduced ability of the conditioned medium to induce osteoblast mineralisation. However, neither addition of TNF-*β* recombinant protein into LNCaP-Id-1-derived conditioned medium, nor addition of TNF-*β* antibodies into PC3-shId-1-derived conditioned medium had any significant effect on the ability of the medium to stimulate osteoclast differentiation (data not shown). These results suggest that modulation of the activity of TNF-*β* in PC3 conditioned medium is not sufficient to alter the ability of the medium to induce osteoclast differentiation. Taken together, these results suggest that overexpression of Id-1 in LNCaP cells results in reduced ability of the cells to induce osteoblast mineralisation through Id-1-mediated reduction of TNF-*β* expression. However, TNF-*β* alone is not sufficient to explain all of the prostate cancer cell-mediated bone cell differentiation resulting from modulating Id-1 expression level.

**Figure 6:**
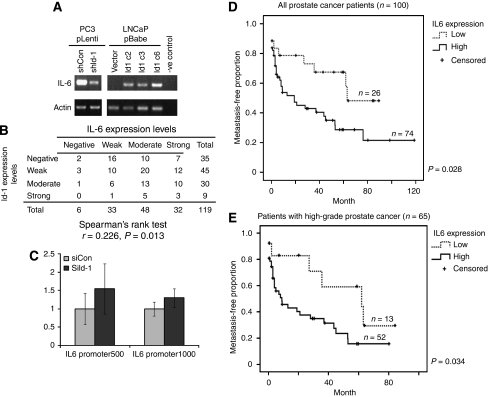
Factors other than TNF-*β* might also be involved in Id-1-modulated prostate cancer-mediated bone cell differentiation. IL-6 mRNA expression is positively correlated with Id-1 expression in LNCaP and PC3 (**A**), and Id-1 is also positively correlated with IL-6 expression in primary prostate specimens (**B**). (**C**) Promoter assay shows that IL-6 promoter activity was not modulated by reduced expression of Id-1. Columns represent the mean values from three independent experiments±s.d. (**D**) Patients with high level of IL-6 staining in their primary prostate cancer had a higher risk of developing distant metastases. (**E**) A similar result was obtained when only patients with high Gleason score (⩾7) were analysed.

Owing to the fact that TNF-*β* could not completely rescue the Id-1-mediated phenotypes, other factors must also participate. In a preliminary study, we found that IL-6 expression is modulated in cells expressing various levels of Id-1 (Figure 6A). A correlation between the expression of the two factors was revealed when benign prostatic hyperplasia specimens were also included in the analysis (Figure 6B, *r*=0.226, *P*=0.013). To analyse whether Id-1 regulates IL-6 at a transcriptional level, we cloned the promoter of IL-6 into a pGL-3 luciferase reporter plasmid and co-transfected this plasmid with siId-1. However, the promoter activity, within 1000 bp upstream of the transcriptional start site, was not significantly affected upon Id-1 knockdown in the transient transfection (Figure 6C). We speculate that Id-1 might regulate IL-6 expression in an indirect manner rather than affecting its immediate transcription, an area that requires further analysis. As IL-6 is correlated with both Gleason grade of the individual cores (Table 2c) and distant metastasis in our patient cohort, regardless of the Gleason score of the tumours (Figure 6D and E), IL-6 might be one factor that could explain the partial rescue of the Id-1-induced phenotypes by recombinant TNF-*β*.

## Discussion

In this study, we have shown that conditioned medium from LNCaP cells overexpressing Id-1 had a higher ability to stimulate osteoclast differentiation than control, whereas Id-1 knockdown in PC3 cells resulted in reduced ability of the conditioned medium to induce osteoclast differentiation. These results suggest that Id-1 has a role in prostate cancer-mediated osteoclast differentiation. In addition, Id-1 also modulates prostate cancer-mediated osteoblast mineralisation. Medium derived from LNCaP-Id-1 cells had a lower ability to stimulate osteoblast mineralisation when compared with their vector control counterpart. However, the medium derived from PC3-shId-1 is still incapable of inducing osteoblast mineralisation, suggesting that Id-1 might be sufficient to inhibit prostate cancer cell-mediated osteoblast mineralisation, but is not the sole regulator of this event. A possible reason for this might be the presence of other Id proteins within the family in PC3. In fact, Id-2 has also been shown to be upregulated in prostate cancer (Coppe *et al*, 2004; Yuen *et al*, 2006), whereas Id proteins have been shown to co-express and have redundant roles in some cellular contexts (Lyden *et al*, 1999).

In search of downstream targets of Id-1 involved in the observed phenotypes, we tested the expression levels of several secretory factors that are known to modulate osteoblast/osteoclast activities using semiquantitative RT–PCR. We found that TNF-*β* might be a downstream target of Id-1. Knowing that Id-1 binds to other helix-loop-helix transcription factors to inhibit their ability to bind DNA and regulate transcription, we proceeded to study, using luciferase reporter assay, whether modulating Id-1 expression could affect the promoter activity of TNF-*β*. We found that overexpression or knockdown of Id-1 in LNCaP cells resulted in reduced and enhanced TNF-*β* promoter activity, respectively. We also analysed the expression levels of TNF-*β* using immunohistochemistry in 110 human prostate cancer specimens for which Id-1 expression status was already known (Yuen *et al*, 2006). We found that, in our prostate cancer patient cohort, high-level expression of Id-1 was associated with low TNF-*β* expression, which is consistent with our *in vitro* findings.

Tumour necrosis factor-*β* has been shown to affect the activities of osteoblasts and osteoclasts (Thomson *et al*, 1987; Frost *et al*, 1997). After confirming the link between Id-1 and TNF-*β*, we went on to study whether TNF-*β* contributes to the Id-1-mediated phenotype. We found that TNF-*β* was responsible for Id-1-mediated inhibition of prostate cancer cell-mediated osteoblast mineralisation, but was not accountable for Id-1-mediated activation of prostate cancer cell-mediated osteoclast differentiation. These results suggest that TNF-*β* has only a partial role in Id-1-modulated prostate cancer cell-mediated bone cell activities.

As TNF-*β* only partially rescued the phenotypes induced by modulating Id-1 in prostate cancer cells, we speculated that secretory factors other than TNF-*β* might also be involved. In a preliminary study, we identified IL-6 as a possible mediating factor, but the mechanisms of Id-1-mediated induction of osteoclast differentiation and inhibition of osteoblast mineralisation are far from clear. To fully understand Id-1-mediated phenotypes, a DNA microarray analysis should be performed that could identify more Id-1 downstream targets. Alternatively, the identification of secretory proteins from the media from cells expressing various levels of Id-1 could also be performed by concentrating the media, analysing the concentrated media in a 2D gel system and identifying the differentially expressed proteins using mass spectrometry. We believe that combinatorial effects from all the Id-1 downstream factors could help to explain the mechanisms of Id-1-mediated prostate cancer-mediated osteoclast differentiation and osteoblast mineralisation.

Recent clinical studies have revealed that bisphosphonate, which functions to block bone destruction, reduces, delays and relieves prostate cancer to bone metastasis (Saad *et al*, 2002, 2004; Saad, 2005). These results suggest that osteoclast-driven bone resorption is an important target for treatment of prostate cancer to bone metastasis. Most prostate cancer to bone metastases are osteoblastic in nature (Koeneman *et al*, 1999). The reason for effective treatment targeting bone resorption may be mainly because of the osteolytic initial stage during the establishment of the bone metastasis. Recent studies on Wnt signalling have shown important evidence that prostate cancer to bone metastasis might transit from an early osteolytic stage to late osteoblastic stage (Hall *et al*, 2005, 2006, 2008). We found that overexpression of Id-1 in LNCaP cells reduced their ability to stimulate osteoblast mineralisation and enhanced their ability to promote osteoclast differentiation, whereas Id-1 knockdown in PC3 cells reduced their ability to stimulate osteoclast differentiation. These results suggest that Id-1 might have an important role in promoting prostate cancer-mediated osteoclast differentiation, and that it could be a target to inhibit prostate cancer to bone metastasis.

In addition, the cell–cell interactions in prostate metastasis in the bone environment are extremely complex. Prostate cancer cells, osteoblasts and osteoclasts interact with each other in the micro-environment and therefore, prostate cancer cells might stimulate osteoblasts to stimulate osteoclasts and *vice versa*. This study did not focus on how these three-dimensional interactions could be modulated by Id-1. A transwell system containing osteoclasts in the top layer and osteoblasts in the bottom layer with conditioned medium from prostate cancer cells might help to elucidate the interaction between these three cell types.

In conclusion, Id-1 has already been shown to promote proliferation, invasion and survival of prostate cancer cells. This study has identified a new role of Id-1 in prostate cancer to bone metastasis and piloted other studies to analyse whether Id-1 could be used as a prognostic marker and therapeutic target in prostate cancer to bone metastasis.

## Figures and Tables

**Table 1:** Patient clinical and pathological features

	**Number of cases**	**%**	**Median (range)**
*Age*	110		73 (56–94) years
*Gleason score*	110		
GS<7	34	31	
GS⩾7	76	69	
			
*Metastatic status*	110		
Non-metastatic	50	45	
Metastatic	60	55	
			
*Gleason score<7*	34		
Non-metastatic	24	71	
Metastatic	10	29	
			
*Gleason score*⩾*7*	76		
Non-metastatic	26	34	
Metastatic	50	66	
			
*Core Gleason grade*	314		
GS=2	8	2	
GS=3	160	51	
GS=4	100	32	
GS=5	46	15	

**Table 2:** (a) Correlation of Id-1 expression levels and Gleason grade of individual cores; (b) correlation of TNF-*β* expression levels and Gleason grade of individual cores; and (c) correlation of IL-6 expression levels and Gleason grade of individual cores

	**GS=2**	**GS=3**	**GS=4**	**GS=5**
**Gleason grade**	**Number (%)**	**Number (%)**	**Number (%)**	**Number (%)**
*(a)*				
*Id-1 expression score*				
Negative	0 (0)	25 (20)	3 (4)	1 (3)
Weak	5 (63)	53 (43)	27 (32)	10 (27)
Moderate	2 (25)	33 (27)	32 (38)	9 (24)
Strong	1 (13)	12 (10)	23 (27)	17 (46)
				
*(b)*				
*TNF-β expression score*				
Negative	1 (17)	26 (22)	16 (19)	8 (20)
Weak	2 (33)	36 (30)	22 (26)	19 (49)
Moderate	2 (33)	36 (30)	28 (33)	9 (23)
Strong	1 (17)	22 (18)	19 (22)	3 (8)
				
*(c)*				
*IL-6 expression score*				
Negative	4 (67)	66 (54)	18 (27)	10 (27)
Weak	2 (33)	33 (27)	24 (36)	7 (19)
Moderate	0 (0)	19 (15)	20 (30)	15 (41)
Strong	0 (0)	5 (4)	5 (8)	5 (14)

(a) Spearman’s rank test: *r*=0.356, *P*<0.001.

(b) Spearman’s rank test: *r*=−0.40, *P*=0.532.

(c) Spearman’s rank test: *r*=0.317, *P*<0.001.
